# A decrease in spontaneous activity in medial prefrontal cortex is associated with sustained hallucinations in chronic schizophrenia: An NIRS study

**DOI:** 10.1038/s41598-020-66560-2

**Published:** 2020-06-12

**Authors:** Masaya Yanagi, Fumiharu Hosomi, Yoshihiro Kawakubo, Aki Tsuchiya, Satoshi Ozaki, Osamu Shirakawa

**Affiliations:** 1 Department of Neuropsychiatry, Kindai University Faculty of Medicine, Osaka-sayama, Osaka Japan; 2 Izumigaoka Hospital, Izumi, Osaka Japan

**Keywords:** Schizophrenia, Imaging

## Abstract

In functional imaging, accumulating evidence suggests that spontaneous activity decreases during the resting state in the core brain regions of the default-mode network [e.g. medial prefrontal cortex (mPFC)] in schizophrenia. However, the significance of this decreased activity has not been clarified in relation to its clinical symptoms. In this study, near-infrared spectroscopy (NIRS), which is a simple imaging modality suitable for resting state paradigm, was used to evaluate the intensity of the spontaneous activity during the resting state in chronic schizophrenia. Consistent with previous findings of fMRI studies, spontaneous activity decreased in the mPFC of patients with schizophrenia. In addition, the decreased spontaneous activity was associated with severe hallucinations in this region where reality monitoring is fundamentally engaged. These results may encourage additional application of NIRS with the resting state paradigm into daily clinical settings for addressing the broad phenotypes and unstable course of schizophrenia.

## Introduction

Since default-mode activity was termed for the brain regional activity that is enhanced during the resting state^1,2^, numerous research fields have been opened up to identify the significance of this activity in human brains and brain diseases^3–5^. The default-mode activity is characterized by spontaneous low-frequency (<0.1 Hz) fluctuations of the brain blood flow^6^. Such fluctuations are synchronized among specific brain regions called the default-mode network whose core regions consist of the medial prefrontal cortex (mPFC) and PCC/precuneus^1,4^. While most of the recent studies about disturbances of this activity have examined the impact on the network changes in psychiatric illnesses, less attention has been paid to quantify the intensity of this activity, which may provide an additional valid marker for mental illnesses.

An index to measure the intensity of the resting-state activity in functional MRI (fMRI) is the amplitude of low-frequency fluctuations (ALFF), which appear dominantly in the core regions of the default-mode network^7,8^. The ALFF indicates the sum of the power across low-frequency range (0.01–0.08 Hz) fluctuations of the brain blood flow, whereas the fractional ALFF (fALFF) indicates the ratio of the ALFF to the total power. Although previous fMRI studies have shown that both the ALFF and fALFF have reliable signals from the grey matter of the brain, the ALFF is believed to be more sensitive to the differences among groups and individuals owing to its higher test-retest reliability^8^. On the other hand, the fALFF is reported to generate lesser noise from physiological sources^7,8^. Therefore, it is recommended to use both analyses to maximize the reliability of examining spontaneous regional brain activity during the resting state^8^.

Several lines of evidence using fMRI suggest that the ALFF or fALFF is decreased during the resting state in the core regions of the default-mode network in schizophrenia^9–16^. However, the extended analysis to explore the relevance of the decreased activity with clinical phenotypes of schizophrenia has shown inconsistent results. Among the five studies that reported the reduction of the ALFF or fALFF in the mPFC of schizophrenia^10,11,14–16^, one reported that ALFF was associated with a general severity of the disease^15^, and another reported an association between fALFF and disorganized symptoms^16^. Another study also reported that the decreased ALFF was recovered with the improvement of positive symptoms in the first episode of schizophrenia^11^. However, the other two studies reported no association between the ALFF and clinical symptoms^10,14^. To overcome such a complexity of pathophysiology in the broad phenotypes of schizophrenia, simple neuroimaging modalities suitable for measurements in routine clinical psychiatry may be warranted.

Near-infrared spectroscopy (NIRS) is a non-invasive imaging device that is easy to use and economically efficient. It demands less physical constraint due to the tolerance for small movements, and measurements can be taken without the acoustic scanner noise that is inevitable in fMRI^17,18^. Because these advantages can provide relatively relaxed circumstances for examinees, NIRS is suitable for resting-state measurements. In addition, test–retest studies have validated the stability of NIRS measurements in the brain network during the resting state^19–21^. Our previous study using wearable NIRS examined the resting-state activity in the mPFC as a region of interest (ROI) and detected a decrease of the fALFF in patients with schizophrenia^22^. However, the significance of the decreased activity in relation to the clinical symptoms of schizophrenia has not been elucidated. This study analyses the ALFF and fALFF using NIRS systematically on the individual channels in the prefrontal regions. It investigates possible associations between the ALFF/fALFF and clinical symptoms in patients with schizophrenia.

## Results

### Alteration of the frontal ALFF/fALFF in patients with schizophrenia

**Figure 1:**
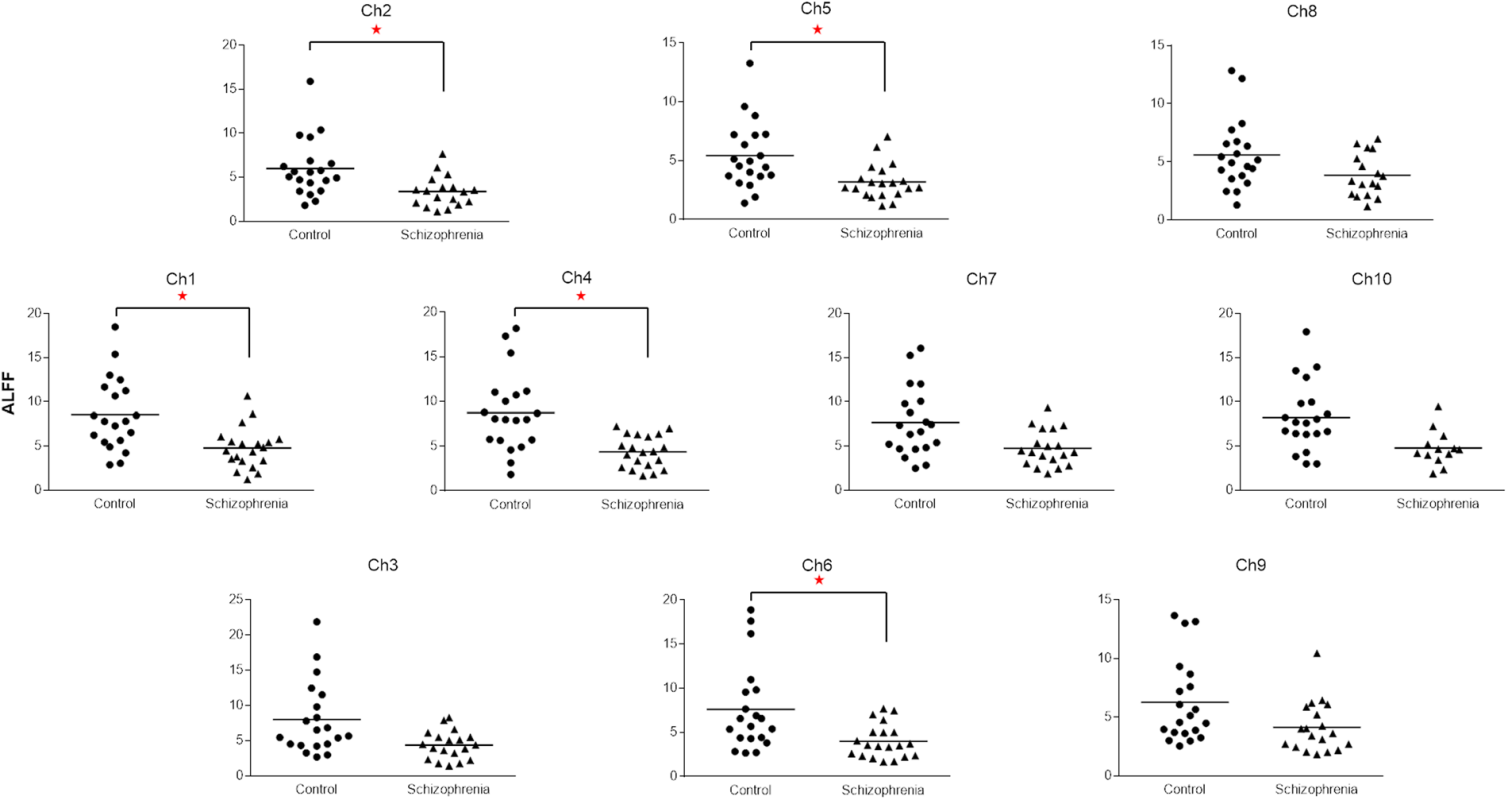
Comparison of the ALFF between patients with schizophrenia and healthy controls on 10 channels of NIRS. The ALFF was significantly decreased on ch1, ch2, ch4, ch5, and ch6 of patients with schizophrenia. *p ≤ 0.005.

Compared with the control subjects, the ALFF was significantly decreased in patients with schizophrenia on five channels [ch1 (control 8.58 ± 0.93, schizophrenia 4.77 ± 0.52, t = 3.6, p = 0.001), ch2 (control 6.00 ± 0.74, schizophrenia 3.38 ± 0.41, t = 3.0, p = 0.005), ch4 (control 8.74 ± 0.98, schizophrenia 4.37 ± 0.40, t = 4.1, p = 0.0002), ch5 (control 5.43 ± 0.64, schizophrenia 3.19 ± 0.34, t = 3.1, p = 0.004), ch6 (control 7.61 ± 1.09, schizophrenia 3.996 ± 0.43, t = 3.1, p = 0.004)], but not on the other channels (p > 0.006) (Fig. 1).

**Figure 2:**
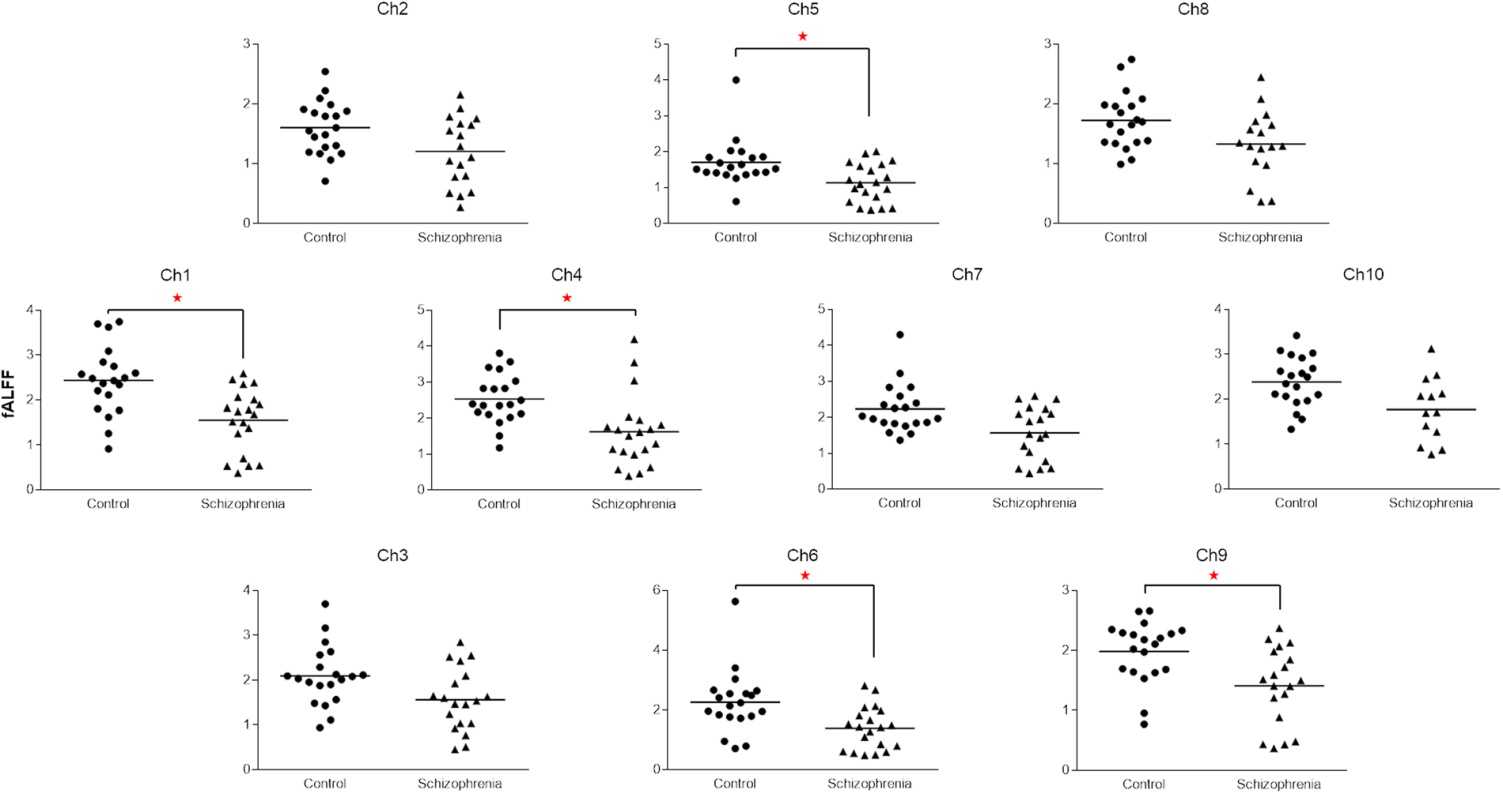
Comparison of the fALFF between schizophrenia patients and healthy controls on 10 channels of NIRS. The fALFF was significantly decreased on ch1, ch4, ch5, ch6, and ch9 of patients with schizophrenia. *p ≤ 0.005.

The fALFF was significantly decreased in patients with schizophrenia on five channels [ch1 (control 2.44 ± 0.17, schizophrenia 1.56 ± 0.16, t = 3.8, p = 0.0005), ch4 (control 2.53 ± 0.15, schizophrenia 1.63 ± 0.22, t = 3.3, p = 0.002), ch5 (control 1.70 ± 0.14, schizophrenia 1.13 ± 0.12, t = 3.0, p = 0.004), ch6 (control 2.26 ± 0.24, schizophrenia 1.39 ± 0.16, t = 3.1, p = 0.004), ch9 (control 1.98 ± 0.11, schizophrenia 4.41 ± 0.15, t = 3.1, p = 0.004)], but not on the other channels (p > 0.006) (Fig. 2).

**Figure 3:**
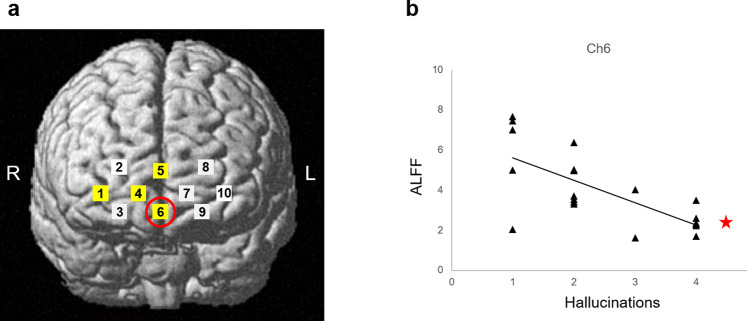
(**a**) Locations for 10 channels of NIRS. The channels where both the ALFF and fALFF showed significant decreases in schizophrenia are highlighted in yellow. The ALFF was significantly associated with severity of hallucinations on the channel circled in red. (**b**) A significant association between the ALFF and severity of hallucinations on ch6 (*p = 0.001).

As such, both the ALFF and fALFF commonly showed significant decreases on four channels (ch1, ch4, ch5, and ch6) in patients with schizophrenia. These channels are highlighted in yellow in Fig. 3a.

### Association of the ALFF/fALFF with the clinical symptoms

In the multiple regression analysis for detecting clinical relevance with decreased ALFF or fALFF in schizophrenia, the ALFF was found to be significantly associated with hallucination scores of CRDPSS on ch6 (β = –0.69, P = 0.001) (Fig. 3b). No other significant association of the clinical variables were observed with the ALFF (p > 0.01) nor with the fALFF (p > 0.01) on any channel.

## Discussion

This is an initial study to show the applicability of NIRS in detecting the significance of decreased ALFF in the symptomatology of schizophrenia. Both the ALFF and fALFF revealed significant decreases in the patients on four channels, which were annotated in the anterior part of the mPFC (ch5 and ch6) and the extended area to the right frontal pole (ch1 and ch4). These results are consistent with those of previous fMRI studies that have shown a decreased ALFF or fALFF in the mPFC of schizophrenia^10,11,14–16^, though NIRS is technically unable to precisely delineate the affected brain region. In the region of ch6 located at the ventral part of the anterior mPFC, a significant association emerged between the ALFF and severity of hallucinations that was evaluated based on the patients’ behaviours, which were influenced by their persistent auditory hallucinations. This finding agrees with the fMRI study which reported an association of improvement of positive symptoms with the restoration of the decreased ALFF in the mPFC^11^. Combined with our results, the decreased ALFF may be continuously observed in the mPFC of patients with schizophrenia who have sustained severe positive symptoms, such as hallucinations.

While previous neuroimaging studies have shown that hyperactivity is observed in the sensory cortex during hallucinatory experiences^23–25^, task-induced brain activity changes validate the mPFC as the additional domain of the hallucinations in schizophrenia^23,25,26^. Psychological fMRI studies have shown that the mPFC is involved in self-monitoring^27^ and that its anterior part specifically works for reality monitoring, which discriminates between self-generated and externally presented information^28^. Additionally, reduced activities have been reported in the mPFC during reality-monitoring tasks in patients with schizophrenia^29–31^. Based on these findings, it is hypothesized that hyperactivity in the sensory cortex accompanied by hypo-activation of the mPFC results in the impairment of reality monitoring, leading to the failure to recognize sensory activities as self-generated and, ultimately, hallucinatory experiences in schizophrenia^28^.

Another line of evidence suggests that the default-mode activity mediates self-referential mental activity. Although the default-mode network is activated during the resting state, it is more potently activated during self-referential tasks^2,4^. Considering that the mPFC is a core brain region of the default-mode network and that the anterior mPFC is involved in reality monitoring among self-referential processing^27^, a reduction in the default-mode activity of the anterior mPFC reflects a weakened reality monitoring in schizophrenia. Taken together with the link between impaired reality monitoring and hallucinations^28^, these conjectures support our results in terms of the association between the severity of hallucinations and reduced spontaneous activity during the resting state in the anterior mPFC in schizophrenia. In short, a decline in the spontaneous activity of the anterior mPFC may be a basis for the weakened reality monitoring that may misattribute self-generated sensory activities as hallucinations.

In the present work, we focused on a limited group of patients with schizophrenia to address the heterogeneous nature of the disease. Because patients with schizophrenia usually have unstable disease courses, chronic patients at a relatively stable phase were recruited. These were hospitalised cases with apparent social functioning deficits who would be probably categorized as psychosis biotype 1, the subgroup typically consisting of deteriorated cases of schizophrenia, as defined in previous studies^32,33^. In addition, only male subjects were examined to reduce clinical variability. Our finding of the significance of decreased ALFF in this adjusted group may encourage the application of NIRS analysis to follow-up studies with larger cohorts to address the broad variety of phenotypes in schizophrenia.

There are some limitations associated with this study. First, NIRS can only measure the cortical surface activity^18,34^, and NIRS signals include contamination of peripheral hemodynamic factors, such as skin perfusion, even in the limited frequency range of blood flow fluctuations for ALFF (0.01–0.08 Hz)^35^. Second, the possible influence of antipsychotic medication cannot be fully excluded on ALFF and fALFF. Although the chlorpromazine-equivalent antipsychotic dose was not significantly associated with the ALFF nor fALFF on any channels, the sample size of our cohort was too small to manage the variation of the antipsychotics. Third, some probe errors were noted on the channels around the side edge of the headset of the wearable NIRS in patients with schizophrenia. This may be due to i) their body motions, and/or ii) their unfitness to the semi-structured headset, which possibly accounts for the minor physical anomalies of the craniofacial measurements that have been reported in schizophrenia^36–39^.

In conclusion, the results of the current study suggest that NIRS is a valid modality to examine the intensity of resting-state activity, such as the ALFF, for evaluating the pathophysiology of schizophrenia. The resting-state paradigm has the advantage of allowing the assessment of patients who may have difficulties adhering to attention-demanding tasks, as is often the case with schizophrenia patients. Given that NIRS is a safe and convenient modality that can offer comfortable circumstance for neurophysiological measurements, its further clinical application should be explored within the paradigm of resting state for psychiatry diseases, such as schizophrenia.

## Methods

### Subjects

Twenty hospitalised male patients (25–68 years, mean ± SEM = 50.6 ± 3.0) with chronic schizophrenia and their case-matched healthy controls (26–66 years, 50.1 ± 2.5) participated in this study. All of them were right-handed. Each patient was diagnosed by two or more psychiatrists on the basis of the DSM-5 criteria for schizophrenia, and the diagnosis was verified based on detailed clinical observations during hospitalisation. All the patients were medicated with antipsychotics at doses that had been largely unchanged for> 3 months because of the persistent symptoms accompanying their chronic disease. Three patients were medicated using the first generation antipsychotics, seven using the second generation, and 10 using both. None had a history of substance/alcohol abuse. The patients were having apparent deficits in social functioning based on the Global Assessment of Functioning (GAF) score of ≤35. The clinical variables of the patients were as follows: GAF score, 24.2 ± 1.4; illness duration, 27.9 ± 3.5 years; onset age, 23.9 ± 1.6 years; and chlorpromazine-equivalent antipsychotic dose, 804.5 ± 143.7 mg/day. The patients’ symptoms were assessed based on the *Clinician-Rated Dimensions of Psychosis Symptom Severity* (CRDPSS)^40^, which is an eight-item scale for dimensional assessment proposed in the DSM-5 to objectively rate the severity of the primary symptoms of psychosis, by two research psychiatrists who were blinded to the NIRS data, along with the information obtained from the clinical psychiatrist in charge and the ward nurses. The mean scores of the CRDPSS among the patients were as follows: hallucinations (2.5 ± 0.3), delusions (2.6 ± 0.2), disorganized speech (3.0 ± 0.2), abnormal psychomotor behaviour (2.5 ± 0.2), negative symptoms (3.3 ± 0.2), impaired cognition (3.2 ± 0.2), depression (0.9 ± 0.1), and mania (0.3 ± 0.2). The controls had no history of neurological or psychiatric disorders. A complete description of the study was provided, and written informed consent was obtained from all the subjects. The study was approved by the Ethics Committee of the Kindai University Faculty of Medicine, and carried out in accordance with the ethical principles of the Declaration of Helsinki and its later amendments.

### Near-infrared spectroscopy

NIRS measurements were performed using a 10-channel wearable NIRS device (WOT-100 system; Hitachi High-Technologies Corporation, Tokyo, Japan), as previously described^22,41,42^. Briefly, the device measured relative changes in oxygenated-(oxy-) and deoxygenated-(deoxy-) hemoglobin (Hb) concentrations using two wavelengths (705 and 830 nm) with a sampling rate of 200 ms. Optical data were analysed using the modified Beer–Lambert law to calculate the signals reflecting changes in Hb levels expressed as arbitrary units (mM–mm)^41,42^. The NIRS probe unit has a 2 × 4 alternating arrangement of irradiation and detection positions. The distance between the pairs of emission and detector probes was set at 30 mm, and the measurement area between the probes was defined as a channel. The lowest probes were positioned along the Fp1–Fp2 line, according to the International 10–20 system^41,43^. The arrangement of channels covered the entire forehead to monitor the activation in the prefrontal regions^41,42^. Three-dimensional coordinates of the channels were obtained using a three-dimensional digitizer (Patriot, POLHEMUS, Inc., Colchester, Vermont, USA), as previously described^22^. The estimate for the spatial registration of the channels using the probabilistic-determination method^43,44^ was mapped onto the Montreal Neurological Institute space based on NIRS-SPM^45,46^ (http://bispl.weebly.com/nirs-spm.html#/) (Fig. 3a). A previous NIRS study of the resting-state paradigm was referenced to designate the mPFC region for the mapped channels^47^.

### Resting-state paradigm

The NIRS signals were acquired during the resting state as previously described^22^. The subjects were seated in a comfortable chair in a silent room to achieve a resting-state condition. They were instructed to focus on the central fixation point displayed on a monitor during the NIRS measurement while keeping their eyes open and remaining still. The NIRS measurement commenced when the subject continually focused on the fixation point on the monitor for 3 min while at rest. The resting paradigm was of relatively short duration, thus enabling patients to continue following the instructions without falling asleep.

### Data analysis

The time courses of oxy- and deoxy-Hb signals were plotted using the BRainSuite_Analyzer (BRSystems. Inc., Kanagawa, Japan) with low pass filter (<0.1 Hz) to eliminate physiological noises, such as those of the heartbeat, respiration, and quick body movements^35,48^. Relative changes in oxy-Hb signals were analysed according to a previous report that demonstrated a strong correlation between oxy-Hb NIRS measurements and blood oxygenation level-dependent signals measured using fMRI^49^. The ALFF and fALFF were analysed using the ALFF/fALFF software (BRSystems Inc.; Brainsuit ALFF; Kanagawa, Japan) according to previous reports^8,50^. This software first transformed the time series of the NIRS data to a frequency domain using the fast Fourier transform, and the power spectrum was obtained for each channel. Then, the square root was calculated at each frequency of the power spectrum. The ALFF was calculated as the sum of the square root across the low-frequency range (0.01–0.08 Hz), and the value of the ALFF was divided by the square root across the total frequency range between 0 and 0.25 Hz to calculate the fALFF.

### Statistics

The ALFF and fALFF were compared between schizophrenia and control groups using the Student’s t-test on the 10 individual channels of NIRS. For the channels where significant changes of the ALFF/fALFF were observed in the schizophrenia group, stepwise multiple regression analysis was conducted to investigate the association between the ALFF/fALFF and clinical variables, including the CRDPSS scores. The ALFF or fALFF was analysed as the dependent variable, and the independent variables were set with clinical variables, such as CRDPSS scores, GAF score, current age, onset age, illness duration, and the chlorpromazine-equivalent antipsychotic dose. The threshold for the significance of p-values was set at 0.005 (0.05/10) by the Bonferroni correction because 10 channel regions were measured in this study. All statistical tests were two-tailed and were conducted using GraphPad Prism 6.0 for Windows version 6.07 (GraphPad Software, Inc., La Jolla, CA, USA) or SPSS version 25.0 (IBM Inc., New York, USA).

### Exclusion of probe error channels

Within the 10 channels measured using NIRS, those judged to be probe errors by the WOT-100 software were excluded from the analysis for each subject. According to this criterion, ch2 was excluded for two patients; ch3, ch7, and ch9 were excluded for one patient; ch8 was excluded for three patients; and ch10 was excluded for seven patients with schizophrenia. There were no probe errors in the control subjects. Because the number of the probe errors on ch10 was significantly different between the schizophrenia and control groups (Fischer’s exact test; p = 0.008), we eliminated the whole dataset of ch10 from the analysis. Notably, ch4, ch5, and ch6 were intact for all patients with schizophrenia and the control subjects.

## Data Availability

The datasets generated and/or analysed during the current study are available from the corresponding author on reasonable request.
